# Effect of Tobacco Cessation Aids on Smoking Cessation and Duration of Abstinence : a French Population-Based Study

**DOI:** 10.1192/j.eurpsy.2022.936

**Published:** 2022-09-01

**Authors:** M. Fekom, V. Nguyen-Thanh, R. Andler, G. Quatremere, R. Guignard, M. Melchior

**Affiliations:** 1 INSERM, Sorbonne University, Team Of Social Epidemiology (eres), Pierre Louis Institute Of Epidemiology And Public Health (iplesp), Paris, France; 2 Santé Publique France, Public Health, Saint Maurice, France

**Keywords:** Pregnancy, cohort study, ADHD symptoms, Addiction

## Abstract

**Introduction:**

Although smoking prevalence has been decreasing worldwide, sustained tobacco cessation remains a challenging goal for many smokers. Nicotine replacement therapy (NRT) products remain among the most widespread type of cessation tobacco aids, along with the more recently introduced electronic cigarette, the efficiency of which is still a matter of debate in the public health community.

**Objectives:**

This study aims to contribute to the ongoing discussion about effective ways of encouraging tobacco cessation and in particular evaluating the role of the two aforementioned tobacco cessation aids with regard to lasting smoking abstinence in real-life settings.

**Methods:**

The study is based on the French 2017 Health Barometer, a cross-sectional survey conducted by Santé Publique France. Two distinct outcomes related to tobacco cessation were used: smoking status at 6 months follow-up (yes vs. no) and the duration of smoking abstinence. These two study outcomes were examined respectively among N1 = 2783 and N2 = 1824 participants. All results were weighted based on inclusion probability weights and controlled for propensity scores via overlap weighting (OW), which is appropriate when exposure groups are disparate.

**Results:**

After adjusting on potential confounders, tobacco cessation at 6 months remains significantly associated with e-cigarette use (OR: 1.50 (1.12-1.99)) and e-cigarette use combined with NRT (OR:1.88 (1.15-3.07)). This association did not reach statistical significance in the long-term analysis, nor did the results of NRT use alone in both analyses.

**Conclusions:**

Overall, while electronic cigarette use alone and combined with NRT is associated with an increase in the likelihood of smoking cessation, the long-term effects are probably limited.

**Disclosure:**

No significant relationships.

